# Isolation and Characterization of a Novel Porcine Teschovirus 2 Strain: Incomplete PERK-Mediated Unfolded Protein Response Supports Viral Replication

**DOI:** 10.3390/v17091200

**Published:** 2025-08-31

**Authors:** Xiaoying Feng, Yiyang Du, Yueqing Lv, Xiaofang Wei, Chang Cui, Yibin Qin, Bingxia Lu, Zhongwei Chen, Kang Ouyang, Ying Chen, Zuzhang Wei, Weijian Huang, Ying He, Yifeng Qin

**Affiliations:** 1 Laboratory of Animal Infectious Diseases and Molecular Immunology, College of Animal Science and Technology, Guangxi University, Nanning 530004, China; 17758620381@163.com (X.F.); 2025207021@stu.njau.edu.cn (Y.D.); 16638404739@163.com (Y.L.); sally2404044152@163.com (X.W.); 15128191582@163.com (C.C.); ouyangkang@gxu.edu.cn (K.O.); yingchen@gxu.edu.cn (Y.C.); zuzhangwei@gxu.edu.cn (Z.W.); huangweijiang-1@163.com (W.H.); 2 Guangxi Zhuang Autonomous Region Engineering Research Center of Veterinary Biologics, Guangxi University, Nanning 530004, China; 3 Guangxi Key Laboratory of Animal Breeding, Disease Control and Prevention, Nanning 530004, China; 4 Guangxi Veterinary Research Institute, Nanning 530004, China; qinyibin5188@163.com (Y.Q.); lubingxia13@163.com (B.L.); chen_zhong-wei@163.com (Z.C.); 5 Guangxi Key Laboratory of Veterinary Biotechnology, Nanning 530001, China; 6 Key Laboratory of China (Guangxi)-ASEAN Cross-Border Animal Disease Prevention and Control, Ministry of Agriculture and Rural Affairs of China, Nanning 530022, China

**Keywords:** porcine teschovirus, isolation, genomic analysis, unfolded protein response, PERK pathway

## Abstract

Porcine Teschovirus (PTV) is a highly prevalent pathogen within swine populations, primarily associated with encephalitis, diarrhea, pneumonia, and reproductive disorders in pigs, thereby posing a significant threat to the sustainable development of the pig farming industry. In this study, a novel strain of PTV was isolated from the feces of a pig exhibiting symptoms of diarrhea, utilizing PK-15 cell lines. The structural integrity of the viral particles was confirmed via transmission electron microscopy, and the viral growth kinetics and characteristics were evaluated in PK-15 cells. High-throughput sequencing facilitated the acquisition of the complete viral genome, and subsequent phylogenetic analysis and full-genome alignment identified the strain as belonging to the PTV 2 genotype. Further investigation revealed that infection with the PTV-GXLZ2024 strain induces phosphorylation of the eukaryotic translation initiation factor 2α (eIF2α) in PK-15 cells, indicating activation of the unfolded protein response (UPR) through the PERK pathway, with minimal involvement of the IRE1 or ATF6 pathways. Notably, ATF4 protein expression was progressively downregulated throughout the infection, while downstream CHOP protein levels remained unchanged, indicating an incomplete UPR induced by PTV-GXLZ2024. Furthermore, PERK knockdown was found to enhance the replication of PTV-GXLZ2024. This study provides critical insights into the molecular mechanisms underlying PTV pathogenesis and establishes a foundation for future research into its evolutionary dynamics and interactions with host organisms.

## 1. Introduction

Porcine Teschovirus (PTV), a member of the Picornaviridae family, possesses a single-stranded, positive-sense RNA genome approximately 7.5 kilobases in length [1]. The viral genome contains a 5′ untranslated region with an internal ribosome entry site (IRES) that facilitates cap-independent translation initiation, followed by a single open reading frame encoding a polyprotein [2]. The genome encodes a polyprotein that undergoes autocatalytic proteolysis, resulting in the production of multiple functional proteins, including structural proteins (VP1, VP2, VP3, VP4) and non-structural proteins (2A, 2B, 2C, 3A, 3B, 3C, 3D). Some strains may also encode an L protein [3,4], the functional role of which remains inadequately understood. The structural proteins self-assemble into an icosahedral capsid essential for host cell recognition and entry, while the non-structural proteins coordinate viral replication and host immune modulation [5]. Genetic diversity is a hallmark of PTVs, with current ICTV classification recognizing two species [2,6,7]: Teschovirus A (22 genotypes) and Teschovirus B (PTV 19, 21 and 22) [8,9,10,11]. Genotype-dependent virulence varies markedly—from the highly neurovirulent PTV-1 (Teschen disease agent) causing fatal polioencephalomyelitis [12] to the attenuated PTV-2 (Talfan strain) associated with subclinical infections. While vaccination controls Teschen disease in Europe, emerging strains in Asia exhibit novel pathogenicity patterns whose mechanisms remain elusive [13,14,15].

As a member of the Picornaviridae family, PTV replication likely induces endoplasmic reticulum (ER) stress through two mechanisms: first, the high demand for viral protein synthesis; second, the remodeling of the ER membrane to form replication complexes. The unfolded protein response (UPR) is considered the primary regulatory mechanism of endoplasmic reticulum (ER) stress, not only regulating protein homeostasis but also interacting functionally with cellular autophagy [16]. Specifically, activation of the UPR induces selective autophagy to degrade misfolded proteins accumulated in the ER lumen, thereby alleviating ERS and promoting cell survival [17,18]. Viral infection can activate the conserved cellular pathways mediated by sensors IRE1α, PERK, and ATF6—collectively known as the UPR—which regulate protein homeostasis [19,20,21,22]. Previous studies have established that the UPR plays a critical role in viral replication and pathogenicity [23,24,25]. However, despite the clinical significance of PTV and the absence of effective therapeutics, no studies have delineated how PTV manipulates UPR pathways.

This study aims to characterize the complete genome of a novel PTV 2 strain and elucidate its interaction with the UPR pathway, laying the foundation for investigating how ERS regulation affects PTV replication. Our findings provide insights into the genetic evolution of PTV and its interactions with host cells, establishing a foundation for further research into its pathogenic mechanisms and the development of antiviral strategies.

## 2. Material and Methods

### 2.1. Virus Detection and Isolation

In February 2024, ten fecal samples from diarrheic pigs on a commercial farm in Liuzhou, China, were homogenized in phosphate-buffered saline (PBS, pH 7.4) at a 1:10 (*w*/*v*) ratio. After centrifugation at 8000× *g* for 10 min at 4 °C, clarified supernatants were subjected to total RNA extraction using TRIzol Reagent (Invitrogen, Carlsbad, CA, USA) following the manufacturer’s protocol. Subsequently, the extracted RNA was reverse-transcribed to generate cDNA by using the RevertAid First Strand cDNA Synthesis Kit (ThermoFisher, Whaltam, MA, USA). The presence of porcine deltacoronavirus (PDCoV), porcine astrovirus (PAstV), porcine epidemic diarrhea virus (PEDV), porcine sapelovirus (PSV), and PTV was analyzed via polymerase chain reaction (PCR) using specific primers, as detailed in Table 1. The PCR conditions were as follows: 95 °C for 3 min, followed by 35 cycles of 94 °C for 30 s, 58 °C for 30 s, and 72 °C for 60 s, with a final extension at 72 °C for 5 min. Amplified products were analyzed by 1.5% agarose gel electrophoresis, and the expected DNA fragments were sequenced by Sangon (Shanghai, China).

For virus isolation, PTV-positive fecal supernatants were individually filtered through 0.22-μm membranes (Millipore, Burlington, MA, USA). Filtrates were inoculated onto confluent PK-15 monolayers (ATCC) cultured in Dulbecco’s Modified Eagle Medium (DMEM) supplemented with 10% fetal bovine serum (FBS) at 37 °C with 5% CO_2_. After 1 h adsorption, cells were washed with PBS and maintained in DMEM/2% FBS. Daily microscopic examination documented cytopathic effects (CPE) over 5–7 days. Supernatants from cultures exhibiting CPE were subjected to three cycles of freeze-thawing, centrifuged at 12,000 rpm for 10 min, and passaged blindly three times. A stable CPE was detected in PK-15 cells following viral infection, and the presence of the virus was confirmed via RT-PCR.

### 2.2. In Vitro Growth Characteristics and Virus Morphology Observation

For virus titration, the virus suspension was serially diluted (from 10^−1^ to 10^−9^) in serum-free DMEM and subsequently added to 96-well plates that had been pre-seeded with PK-15 cells. The plates were incubated at 37 °C in a 5% CO_2_ atmosphere for a duration of five days. The number of wells exhibiting cytopathic effects (CPEs) at each dilution was recorded, and virus titers, expressed as the 50% tissue culture infective dose (TCID_50_), were calculated using the Reed and Muench method [31]. To assess the in vitro growth characteristics of the isolated virus, it was inoculated into PK-15 cells, ST cells, and Vero cells at a multiplicity of infection (MOI) of 0.1. Cytopathic effects were monitored at 6, 12, 24, 36, and 48 h post-infection (hpi). To further investigate the in vitro replication characteristics of the isolated strain, the virus was inoculated into PK-15 cells seeded in 12-well plates at an MOI of 0.1. Cell culture supernatants were collected at 6, 12, 18, 24, 48, and 72 hpi, and viral titers at each time point were quantified using the TCID_50_ assay as previously described. A multi-step growth curve was plotted, with the infection time on the *x*-axis and the TCID_50_ values of the virus in the supernatant on the *y*-axis.

To examine the morphology of virus particles via electron microscopy, a 50 mL virus suspension was subjected to centrifugation at 13,000 rpm for 1 h at 4 °C. The resulting supernatant was filtered using a 0.22 μm membrane. Subsequently, a total of 12.5 mL of a 50% PEG-8000 solution—comprising 20 mL of double-distilled water, 0.8766 g of NaCl, and 5 g of PEG-8000—was incrementally added in three portions. This mixture was then agitated at 200 rpm for 12 h at 4 °C. Following this incubation, the mixture underwent another round of centrifugation at 13,000 rpm for 1 h at 4 °C to isolate the precipitate. The resulting pellet was resuspended in 100 μL of PBS, and the resuspended virus particles were subsequently subjected to negative staining and analyzed using a transmission electron microscope (Hitachi, Hitachi, Japan).

### 2.3. Whole Genome Sequencing and Sequence Analysis

To acquire the complete genome sequence of isolated strain, total RNA extracted from small RNA virus isolates was provided to BGI Genomics (Shenzhen, China) for strand-specific library construction and sequencing. Ribosomal RNA-depleted libraries were prepared using the MGIEasy RNA Library Prep Kit with dUTP-based strand marking, followed by 150 bp paired-end sequencing on the DNBSEQ-T7 platform. Bioinformatics analysis included adapter trimming (Trimmomatic v0.39), host genome subtraction (HISAT2 v2.2.1 against Sus scrofa ARS1.2), de novo assembly (SPAdes v3.15.3), and recombination screening (RDP5), achieving >20× coverage depth with Q30 ≥ 85% across all samples. The 5′UTR and 3′UTR of the isolated strain were amplified using the SMART RACE cDNA Amplification Kit (Takara, Dalian, China). The complete genome sequence of the strain was assembled and verified using SnapGene software (version 7.2). Sequence alignment was performed using the DNASTAR software package (version 7.1) and Jalview software (version 2.1).

### 2.4. Phylogenetic and Recombination Analyses

The complete genome sequences of reference PTV strains utilized in this study were sourced from the NCBI database. Phylogenetic trees were constructed based on the entire genome sequences and P1 gene sequences of PTV strains using MEGA software (version 11) employing the neighbor-joining method. To ensure robustness, bootstrap analysis was conducted with 1000 replicates. The resulting phylogenetic trees were visualized via the Chiplot online platform (https://www.chiplot.online/tvbot.html, accessed on 29 June 2025). Recombination analysis of the complete genome of the isolated strain was performed using RDP4 software with seven methods (RDP, GeneConv, Chimaera, MaxChi, BootScan, SiScan and 3Seq), with a maximum acceptable *p*-value of 0.05. This stringent criterion minimizes the risk of false positives. Putative recombinant events were further verified by SimPlot v3.5.1 software [32] with default parameters.

### 2.5. Generation of Polyclonal Antibodies Against the PTV VP1 Protein

To generate polyclonal antibodies against PTV, specific primers were designed to target the VP1 gene of PTV. The forward primer sequence was 5′-CCGGAATTCATGCCTGCTGAGACAGGCTGTGAT-3′, and the reverse primer sequence was 5′-CCCAAGCTTGCTGCAGTGATAATGTTGTCAT-3′, both incorporating *EcoR I* and *Hind III* restriction sites at their 5′ termini. These primers facilitated the PCR amplification of the target gene. The resulting amplified fragment was subsequently cloned into the pET-32a(+) vector to construct the pET32a-VP1 recombinant plasmid. This plasmid was transformed into *Escherichia coli* BL21 (DE3) cells, and expression of the VP1 protein was induced using 0.5 mM isopropyl β-D-1-thiogalactopyranoside (IPTG) at 37 °C for a duration of 10 h. Cell lysis was achieved through the application of a lysis buffer containing lysozyme, followed by mechanical disruption via sonication. Protein expression was verified through Western blot analysis using an anti-His monoclonal antibody. The expressed VP1 protein was subsequently purified using nickel-nitrilotriacetic acid (Ni-NTA) affinity chromatography (Kangwei Century Biotechnology, Taizhou, Jiangsu, China). The protein concentration was determined using a bicinchoninic acid (BCA) assay kit (Takara). The purified VP1 protein, emulsified with Freund’s complete adjuvant, was administered subcutaneously to BALB/c mice at a dosage of 100 µg per mouse. Fourteen days post-initial immunization, two booster doses of the protein, emulsified in Freund’s incomplete adjuvant (100 µg per mouse, administered at one-week intervals), were given. Seven days subsequent to the third immunization, serum samples were collected for the determination of antibody titers via indirect ELISA. In this assay, the purified VP1 protein (at a concentration of 2 µg/mL in 50 mM carbonate buffer, pH 9.6) was coated onto 96-well plates and incubated overnight at 4 °C. Following washing with PBST, the plates were blocked with 5% skim milk for 2 h. Serial dilutions of the serum (ranging from 1:500 to 1:102,400) were then added and incubated at 37 °C for 1 h. After an additional washing step, HRP-conjugated goat anti-mouse secondary antibody (diluted 1:20,000) was added and incubated at 37 °C for 1 h. Following a final wash, the reaction was developed using TMB substrate for 15 min at room temperature, and the optical density was measured at 450 nm after terminating the reaction.

### 2.6. Western Blot and Indirect Immunofluorescence Assays (IFA)

PK-15 cells were infected with the isolated strain at a MOI of 0.01. At 24 h post-infection (hpi), viral protein expression was assessed using Western blot and IFA with a VP1-specific polyclonal antibody developed in our study. For the Western blot analysis, infected cells were collected and lysed, and total proteins were separated via 10% sodium dodecyl sulfate-polyacrylamide gel electrophoresis (SDS-PAGE). These proteins were then electrophoretically transferred onto a polyvinylidene difluoride (PVDF) membrane (Merck Millipore, Billerica, MA, USA). The membrane was probed with the VP1-specific polyclonal antibody as the primary antibody to detect the PTV VP1 protein. Following three washes with Tris-buffered saline containing 0.1% Tween-20 (TBST), the membrane was incubated with a horseradish peroxidase (HRP)-conjugated goat anti-mouse IgG secondary antibody (Proteintech, Wuhan, China) for 1 h at room temperature. Protein bands were visualized using a BeyoECL Plus chemiluminescence kit (Beyotime Biotechnology, Shanghai, China).

For the indirect IFA, infected cells were fixed using 4% paraformaldehyde at 4 °C for 30 min. Subsequently, the cells underwent five washes with PBS containing 0.1% Tween-20 (PBST). To block non-specific binding, the cells were incubated with 1% bovine serum albumin (BSA) in PBST at 37 °C for 2 h. The cells were then exposed to a VP1-specific polyclonal antibody, diluted in the blocking buffer, serving as the primary antibody, and incubated at 37 °C for 3 h. This was followed by incubation with a fluorescein isothiocyanate (FITC)-conjugated goat anti-rabbit IgG (H+L) secondary antibody (Proteintech) at 37 °C for 1 h. After five additional washes with PBST, the nuclei were counterstained with 4′, 6-diamidino-2-phenylindole (DAPI) at a concentration of 1 μg/mL for 10 min. Fluorescence signals were subsequently observed using a Nikon Eclipse Ti fluorescence microscope (Nikon Corporation, Tokyo, Japan).

### 2.7. Development of a qPCR Assay for PTV Viral Quantification

To develop a quantitative PCR (qPCR) assay for the detection of PTV, specific primers targeting the VP1 gene (Table 2) were designed by Tsingke (Beijing, China). Viral RNA extraction and reverse transcription were conducted as outlined in Section 2.1. The target VP1 gene was subsequently amplified via PCR and cloned into the pMD-18T vector (Takara), resulting in the recombinant plasmid pMD-18T-VP1. This plasmid was subjected to serial dilution (ranging from 10^−3^ to 10^−9^) and utilized as a template in a 10 μL reaction system, which comprised 3.5 μL of double-distilled water (ddH2O), 5 μL of 2x Taq Pro Universal SYBR qPCR Master Mix (Vazyme, Nanjing, China), 0.25 μL of each primer, and 1 μL of complementary DNA (cDNA). The qPCR conditions were set as follows: initial denaturation at 95 °C for 30 s, followed by 40 cycles of denaturation at 95 °C for 10 s and annealing/extension at 60 °C for 30 s, with data acquisition occurring during the 60 °C phase. The reactions were executed on a LightCycler^®^ 96 real-time PCR system (Roche, Basel, Switzerland), and the data were processed using LightCycler^®^ 96 Software (version 1.1). The quantification cycles (Cq) were determined utilizing the Roche LightCycler^®^ 480 system.

### 2.8. Analyzing the Activation of UPR Signaling Pathway Induced by PTV Infection

The UPR signaling pathway encompasses three distinct pathways: PERK, ATF6, and IRE1. To investigate PERK and ATF6 pathway activation upon PTV infection, PK-15 cells were infected with the isolated viral strain at a multiplicity of infection (MOI) of 0.01. Thapsigargin (TG, 1 μM) and untreated cells served as positive and negative controls, respectively. At 6, 12, 24, and 36 h post-infection (hpi), the cells were harvested and lysed using RIPA buffer (Solarbio, Beijing, China). Protein concentrations were determined via the bicinchoninic acid (BCA) assay. Subsequently, equal amounts of protein (20 µg per lane) were resolved by 12% SDS-PAGE and transferred onto polyvinylidene difluoride (PVDF) membranes. The membranes were blocked with 5% non-fat milk in Tris-buffered saline with 0.1% Tween-20 (TBST) for 2 h at room temperature, followed by overnight incubation at 4 °C with primary antibodies targeting ATF6, PERK, eIF2α, phospho-eIF2α, ATF4, and CHOP (Proteintech, Wuhan, China). After three washes with TBST, the membranes were incubated with horseradish peroxidase (HRP)-conjugated secondary antibodies (dilution 1:5000) for 1 h at room temperature. Protein bands were visualized using an enhanced chemiluminescence (ECL) detection system (Bio-Rad, Hercules, CA, USA), and band intensities were quantified using ImageJ 1.53t software (National Institutes of Health, USA). Concurrently, cell samples were collected at 6, 12, 24, and 36 h post-infection, and the mRNA expression levels of AT4 and CHOP were assessed via relative quantitative PCR, as detailed in a previous study [33].

To investigate the activation of the IRE1 pathway following PTV infection, cell samples were collected at 6, 12, 24, and 36 h post-infection (hpi). Total RNA was extracted and reverse transcribed into cDNA, as detailed in Section 2.1. The porcine XBP1 was then amplified using the PCR method previously described previously [33]. The resulting PCR products were purified using the QIAEX^®^ II Gel Extraction Kit (Qiagen, Beijing, China). Subsequently, the purified PCR products underwent single digestion with the Pst I restriction enzyme (Takara) at 37 °C for 1 h. The digested products were separated on a 1.5% agarose gel and visualized using a Gel Imaging System (Bio-Rad, USA).

### 2.9. siRNA-Mediated PERK Knockdown

To achieve PERK gene knockdown in PK-15 cells via siRNA technology, a specific siRNA targeting PERK was designed and synthesized by Sangon Biotech (Shanghai, China). The siRNA sequences used were as follows: sense strand, 5′-GGUAAUGCGAGAAGUUAAA-3′; antisense strand, 5′-UUUAACUUCUCGCAUUACC-3′. PK-15 cells were seeded in 12-well plates and transfected with either PERK-specific siRNA or scrambled siRNA (serving as a negative control) using siRNA-mate plus (GenePharma, Suzhou, China), following the manufacturer’s instructions. At 6, 12, and 24 h post-transfection, cells were lysed to collect total proteins. The efficiency of PERK knockdown was assessed through Western blotting analysis using an anti-PERK antibody (Proteintech). To evaluate the impact of the PERK pathway on PTV replication, both PERK-knockdown and scrambled siRNA-transfected control cells were infected with the isolated strain at a multiplicity of infection (MOI) of 0.01. At 24 h post-infection (hpi), total RNA was extracted and reverse-transcribed into cDNA. Viral RNA copy numbers were quantified using qPCR, as established in this study. Concurrently, cell culture supernatants were collected, and viral titers were measured using the TCID_50_ assay, as detailed in Section 2.3.

### 2.10. Statistical Analysis

Statistical analyses were performed with GraphPad Prism 8.0 using two-way ANOVA to evaluate differences among groups. Data are shown as mean ± standard deviation, with significance indicated as follows: *, *p* < 0.05; **, *p* < 0.01; ***, *p* < 0.001; ****, *p* < 0.0001.

## 3. Results

### 3.1. Isolation, Identification, and Growth Characteristics of PTV-GXLZ2024

Fecal specimens from pigs exhibiting diarrhea were analyzed for the presence of several common porcine diarrhea-associated viruses. The results indicated that the fecal sample tested positive for PTV, whereas PDCoV, PAstV, PEDV, and PSV were all negative. To isolate the PTV strain, the PTV-positive fecal samples were inoculated into PK-15 cells. After two passages, significant cytopathic effects (CPE) were observed. The cell culture supernatant was collected, and total RNA was extracted. The successful isolation of PTV was confirmed using the reverse transcription polymerase chain reaction (RT-PCR) method, and the isolated strain was designated as PTV-GXLZ2024. This isolated strain was serially passaged to the 10th generation (F10), achieving a viral titer of 10^6.8^ TCID_50_/mL.

**Figure 1:**
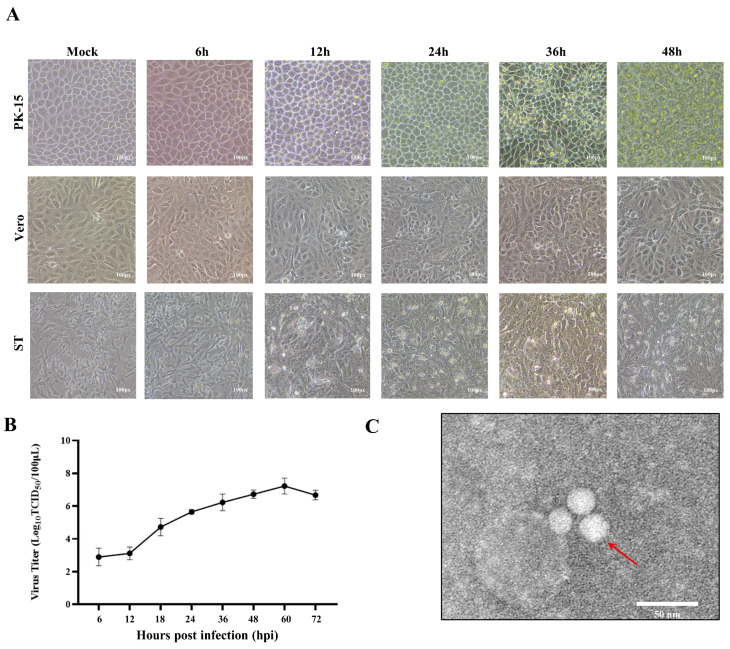
Isolation and Characterization of PTV-GXLZ2024. (**A**) Infection of PTV-GXLZ2024 in different cell lines (PK-15, Vero, and ST cells). (**B**) A multi-step growth curve of the PTV-GXLZ2024 strains were generated in PK-15 cells. (**C**) The morphology of PTV-GXLZ2024 viral particles was examined using electron microscopy.

To evaluate the cellular tropism of the PTV-GXLZ2024 strain, PK-15, Vero, and ST cells were infected with the strain at a MOI of 0.1. In PK-15 cells, pronounced cytopathic effects (CPE), characterized by cell aggregation, rounding, and detachment, were observed at 24 h post-infection (hpi), with significant cell lysis occurring by 36 hpi (Figure 1A). In contrast, the PTV-GXLZ2024 strain did not induce CPE in Vero and ST cells (Figure 1A), and no viral presence was detected in these cell lines, suggesting that PTV-GXLZ2024 does not infect Vero and ST cells. The multi-step growth curve analysis demonstrated that the viral titers of PTV-GXLZ2024 increased steadily from 6 to 60 hpi, peaking at 60 hpi with a maximum viral titer of 10^7.0^ TCID_50_/mL, followed by a gradual decline (Figure 1B). Transmission electron microscopy (TEM) analysis revealed that the PTV-GXLZ2024 strain consists of smooth, spherical, non-enveloped particles with diameters ranging from 25 to 30 nm (Figure 1C).

### 3.2. Comprehensive Genome Sequencing and Sequence Analysis of PTV-GXLZ2024

**Figure 2:**
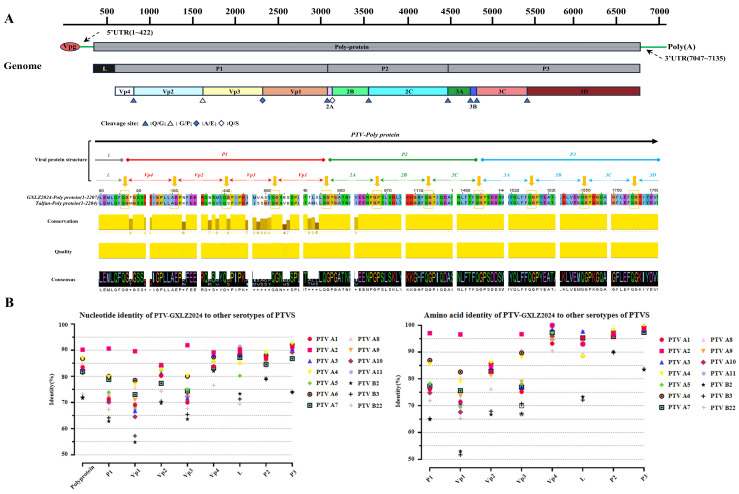
Whole genome sequence analysis of PTV-GXLZ2024. (**A**) Schematic diagram of the PTV-GXLZ2024 genome structure and analysis of polyprotein cleavage sites. (**B**) Sequence and protein homology analysis of PTV-GXLZ2024 compared to different PTV genotypes.

The complete genome sequence of the PTV-GXLZ2024 strain (GenBank accession number: PQ358532.1) was determined using high-throughput sequencing techniques. The 5′ and 3′ untranslated regions (UTRs) were further characterized through the rapid amplification of cDNA ends (RACE) method. The analysis revealed that the total genome length of the PTV-GXLZ2024 strain is 7093 nucleotides (nt), comprising a 5′ UTR (1–422 nt), a 3′ UTR (7047–7093 nt), and a single open reading frame (ORF) spanning 423 to 7046 nt. This ORF encodes a polyprotein consisting of 2207 amino acids, which includes the L gene (423–580 nt), P1 gene (681–3260 nt), P2 gene (3261–4724 nt), and P3 gene (725–7043 nt). The P1 gene encodes the structural proteins VP1, VP2, VP3, and VP4, while the P2 and P3 gene encode the non-structural proteins 2A, 2B, 2C, and 3A, 3B, 3C, 3D, respectively. The cleavage sites are predominantly Q/G, with exceptions at G/P (VP2/VP3) and Q/S (2A/2B), aligning with the characteristics observed in most PTV strains (see Figure 2A).

The results of the homology comparison analysis, as presented in Table 3 indicate that the PTV-GXLZ2024 strain exhibits nucleotide identity ranging from 81.4% to 90.2% and amino acid identity from 79.1% to 93.6% with Teschovirus A strains across the complete genome. Notably, the highest homology is observed with the PTV 2 strains, with nucleotide and amino acid identities reaching 90.2% and 93.6%, respectively. Conversely, the nucleotide and amino acid identities with Teschovirus B strains are significantly lower, at 71.6–73.2% and 51.3–55.9%, respectively. The P1 gene, utilized for genotyping, demonstrates nucleotide identity ranging from 62.8% to 90.6% and amino acid identity from 64.8% to 97.1% with PTV strains of various genotypes, with the highest identity observed with PTV 2 strains (90.6% nucleotide, 97.1% amino acid). Among the structural proteins, VP4 is identified as the most conserved, followed by VP2 and VP3, whereas VP1 is the most variable. The L protein exhibits the highest identity with PTV 11 strains (91.5% nucleotide, 97.7% amino acid), and the P2/P3 proteins show the highest identity with PTV 4 strains, with nucleotide identities of 88.9% and 92.3% and amino acid identities of 98.2% and 99.4%, respectively, as illustrated in Figure 2B.

### 3.3. Phylogenetic and Recombination Analysis of PTV-GXLZ2024

**Figure 3:**
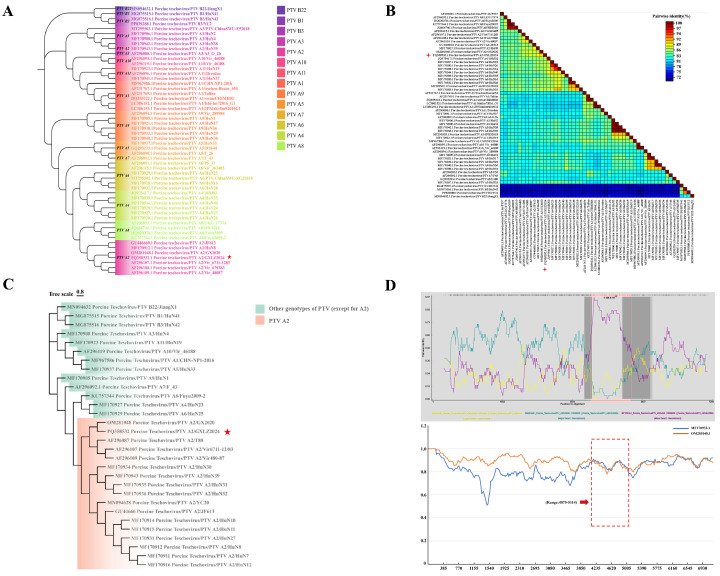
Phylogenetic Analysis and Recombination Prediction of PTV-GXLZ2024. (**A**) A phylogenetic tree was constructed based on the complete genome sequences of PTV, encompassing Teschovirus A and B. The PTV-GXLZ2024 is denoted by red five-pointed stars. (**B**) An analysis of pairwise identity among the whole genome sequences of PTV across different genotypes was performed. (**C**) A phylogenetic tree was constructed based on the full-length VP1 gene of different genotypes of PTV. (**D**) Recombinant analysis of the complete genome of PTV-GXLZ2024 was performed utilizing the RDP4 (upper graph) and Simplot software packages (bottom graph). A recombination event, occurring between nucleotide positions 4075 and 5114, is highlighted by a red box.

To elucidate the evolutionary relationships of PTV-GXLZ2024, a phylogenetic tree was constructed utilizing the complete genome sequence of PTV strains. The analysis indicated that the Teschovirus genus comprises two distinct species: Teschovirus A and Teschovirus B. PTV-GXLZ2024 was categorized within the PTV 2 genotype of Teschovirus A (Figure 3A). Pairwise sequence comparisons demonstrated substantial divergence between Teschovirus A and Teschovirus B, with sequence identity falling below 70%. Within Teschovirus A, PTV 2, 4, 6, and 8 genotypes exhibited close phylogenetic affiliations, characterized by sequence identities of 80% or higher and genetic distances ranging from 0 to 0.29. Notably, PTV-GXLZ2024 exhibited the closest phylogenetic relationship to the GX2020 strain of PTV 2, with a genetic distance of 0.11 and a sequence identity of 90% (Figure 3B). A phylogenetic tree based on the P1 gene was constructed using the maximum likelihood (ML) method (Figure 3C). The results indicated that, consistent with the complete genome phylogenetic tree, PTV-GXLZ2024 clustered within the PTV 2 clade. It exhibited a minimum genetic distance of 0.34 from other genotypes (PTV 6), which exceeds the maximum intra-genotype distance of 0.24, thereby further confirming the classification of the PTV-GXLZ2024 strain into the PTV 2. Recombination analysis revealed a recombination event in the PTV-GXLZ2024 genome within the nucleotide region 4075–5114, with GX2020 (OM281048) and HuN8 (MF170912) identified as the major and minor parental strains, respectively (*p*-value = 1.124 × 10^−17^; Figure 3D).

### 3.4. Preparation of Polyclonal Antibody and Establishment of RT-qPCR Method for PTV-GXLZ2024

**Figure 4:**
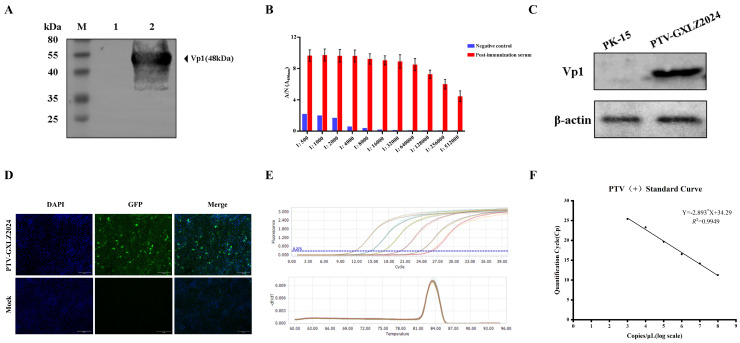
Preparation of PTV-VP1 polyclonal antibodies and establishment of the RT-qPCR detection method. (**A**) Western blot identification of the expression of recombinant VP1 protein. (**B**) Titer determination of the VP1 polyclonal antibody. (**C**,**D**) Verification of the availability of VP1 polyclonal antibodies through Western blot (**C**) and IFA (**D**). (**E**) Amplification curve and Melting curve of the standard plasmid with gradient dilutions (indicated by different colors). (**F**) Standard curve of RT-qPCR for PTV-GXLZ2024.

To generate polyclonal antibodies against PTV, the VP1 gene of the PTV-GXLZ2024 strain was amplified via PCR, cloned into the pET-32a(+) vector, and subsequently expressed in BL21(DE3) cells. SDS-PAGE analysis confirmed the expression of a 48 kDa target protein, consistent with the anticipated size, which was produced as inclusion bodies (Figure 4A). The protein was purified and used to immunize KM mice on three occasions, resulting in a serum antibody titer of 1:512,000 (Figure 4B). The generated VP1 polyclonal antibody demonstrated specific recognition of the PTV-GXLZ2024 strain in PK-15 cells, as evidenced by Western blotting (Figure 4C) and immunofluorescence assay (IFA) (Figure 4D). Concurrently, an RT-qPCR method was developed to specifically detect the PTV-GXLZ2024 strain. The uniform melting curve, devoid of any extraneous peaks, indicates that the RT-qPCR method established in this study exhibits high specificity (Figure 4E) and is suitable for the quantification of PTV copy numbers. A standard curve was constructed using serially diluted recombinant plasmid templates, revealing a linear correlation between Cq values and template copy numbers, with a regression equation of y = −2.8933x + 34.299 (R^2^ = 0.9959) (Figure 4F). The designed primer had good specificity for PTV and did not react with 5 other viruses and negative control, including porcine deltacoronavirus (PDCoV), porcine astrovirus (PAstV), porcine epidemic diarrhea virus (PEDV), porcine sapelovirus (PSV) and Getahvirus (GETV).

### 3.5. Activation of the PERK Pathway by PTV-GXLZ2024 in the UPR

**Figure 5:**
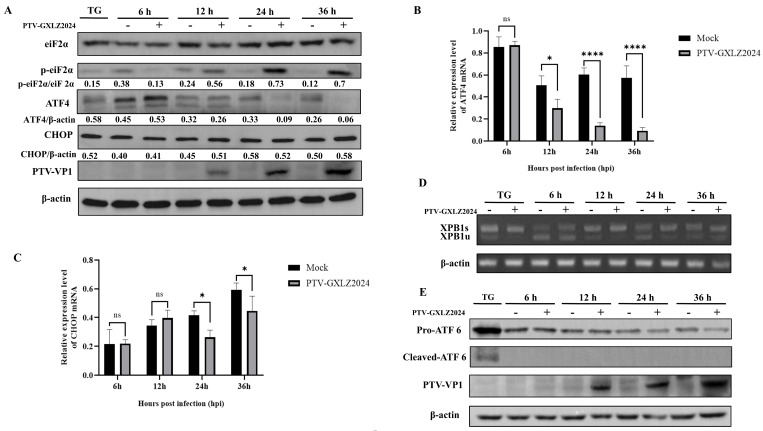
PTV-GXLZ2024 infection induced UPR. (**A**) Western blotting detection of eIF2α, phospho-eIF2α, ATF4, CHOP, VP1, and β-actin protein expression after infection of PK-15 cells with PTV-GXLZ2024 strain. (**B**,**C**) Detection of ATF4 (**B**) and CHOP (**C**) mRNA levels after infection of PK-15 cells with the PTV-GXLZ2024 strain by RT-qPCR. (**D**) Detection of XBP 1 mRNA splicing after PTV-GXLZ2024 infection in PK-15 cells. (**E**) Western blot analysis of ATF6 protein expression in PTV-GXLZ2024-infected PK-15 cells.

The activation of the PERK, IRE1α, and ATF6 signaling pathways following PTV-GXLZ2024 infection was investigated. The results demonstrated a significant increase in the phosphorylation of eIF2α (p-eIF2α) beginning at 12 h post-infection (hpi), with a peak observed at 36 hpi. ATF4 protein levels increased at 6 hpi but were significantly reduced at 24 hpi and 36 hpi, with almost no detectable ATF4 protein expression at 36 hpi (Figure 5A). qPCR analysis revealed that ATF4 mRNA expression levels continuously decreased from 12 hpi to 36 hpi (Figure 5B). The protein expression levels of CHOP, a downstream target of ATF4, remained constant from 6 to 36 hpi (Figure 5A), while its mRNA expression levels were significantly reduced at 24 and 36 hpi (Figure 5C). These findings suggest that PTV-GXLZ2024 infection may activate the PERK pathway via eIF2α phosphorylation but inhibits the activation of downstream molecules ATF4 and CHOP. The study employed RT-PCR in conjunction with enzyme digestion and Western blotting analysis to assess the activation status of the IRE1α-XBP1 and ATF6 signaling pathways. The findings indicated that spliced XBP1 mRNA was absent in PK-15 cells infected with PTV-GXLZ2024 (Figure 5D), suggesting that the IRE1α-XBP1 pathway was not activated following infection with PTV-GXLZ2024. Similarly, Western blotting analysis demonstrated an absence of ATF6 cleavage in PTV-GXLZ2024-infected cells, further implying that the ATF6 pathway was also not activated (Figure 5E).

### 3.6. Si-PERK Facilitates PTV-GXLZ2024 Replication

**Figure 6:**
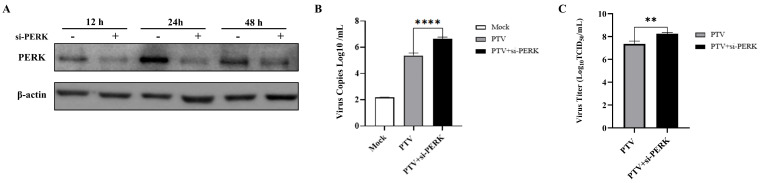
The effects of si-PERK on the replication of PTV-GXLZ2024. (**A**) Western blot analysis of PERK protein expression after treatment with si-PERK. (**B**) Detection of viral copy number of PTV-GXLZ2024 after treatment with si-PERK by RT-qPCR. (**C**) Collection of the supernatant from si-PERK-treated cells infected with PTV-GXLZ2024, and measurement of the virus titer using the TCID_50_ method.

To elucidate the involvement of the PERK-eIF2α-ATF4 pathway in the replication of PTV-GXLZ2024, a PERK-specific siRNA was employed to transiently reduce PERK expression in PK-15 cells. Western blotting analysis confirmed a significant reduction in PERK protein levels 12 h following siRNA transfection (Figure 6A). Quantitative PCR (qPCR) analysis indicated that the siRNA targeting PERK (si-PERK) led to a significant increase in viral mRNA levels relative to the control group (Figure 6B). Additionally, TCID_50_ assays demonstrated that PERK knockdown led to an increase in viral titers to 10^8.25^/mL, which is significantly higher than the control group, which exhibited titers of 10^7.36^/mL (Figure 6C). These findings suggest that PTV-GXLZ2024 infection activates the PERK-eIF2α pathway, and that the knockdown of PERK enhances viral replication.

## 4. Discussion

Porcine Teschovirus (PTV), historically recognized for inducing severe encephalomyelitis in swine during the early 20th century, was initially characterized by the predominance of highly virulent PTV 1 strains associated with elevated mortality rates. Over time, the prevalent circulating strains have transitioned from these highly pathogenic PTV 1 to variants with reduced pathogenicity [34], likely driven by the necessity for enhanced transmission efficiency. Presently, PTV is ubiquitous across pig populations of all ages globally, predominantly resulting in asymptomatic infections. Nonetheless, infections with certain virulent strains can still precipitate reproductive disorders, pneumonia, diarrhea, and encephalomyelitis, with sporadic regional outbreaks documented in Europe and Africa [35]. These observations underscore the necessity for sustained vigilance against the potential threats posed by PTV. A comprehensive understanding of the pathogenic mechanisms of PTV is essential for effective disease control and prevention strategies.

Initially categorized as a genotype of Porcine Enterovirus (PEV), PTV has been predominantly studied for its pathogenic effects on intestinal tissues. Research indicates that PTV initially replicates in the tonsils and intestines upon infection [7]. The intestines, which demonstrate the highest viral titers post-infection, are the earliest sites of viral detection, underscoring their pivotal role in the progression from viral invasion to the onset of symptoms. Extensive viral replication within the intestines may facilitate systemic dissemination to other organs [7]. Although certain virulent strains are capable of infecting the central nervous system (CNS), including the brain and spinal cord, the mechanisms underlying PTV infection in tissues beyond the intestines and CNS remain inadequately understood.

In this study, we successfully isolated a strain of PTV, designated as PTV-GXLZ2024, from diarrheic pig feces using PK-15 cells. Notably, PTV-GXLZ2024 was unable to infect Vero and ST cells, which are typically susceptible to PTV infection. This observation may be attributed to variations in receptor expression or specificity across different cell types. The VP1 protein of picornaviruses is crucial for receptor recognition and cell entry [36], indicating that variations in VP1 among strains may confer distinct receptor specificities. Recent research suggests that nearly all PTV A strains can infect porcine kidney cells, while certain PTV 1 and PTV 2 strains are also capable of infecting ST cells [37], implying a potential correlation between infection tropism and genotype. Conversely, PTV B strains demonstrate an opposite pattern, infecting ST cells but not PK-15 cells [38]. Considering that PTV 1 strains, historically the earliest and most pathogenic PTV variants, exhibit unique tropism, we hypothesize that infection tropism may be linked to evolutionary adaptation and genotype divergence. Future research will focus on the receptor-binding regions of the VP1 protein in PTV-GXLZ2024 to elucidate its mechanisms of cellular tropism.

The PTV-GXLZ2024 strain isolated in this study is classified under PTV 2, a prevalent genotype in China, and demonstrates phylogenetic clustering with the previously identified Guangxi strain MF170912, suggesting a local origin. Notably, the pigs from which the positive fecal samples were obtained exhibited not only diarrhea but also neurological symptoms, with postmortem examinations revealing lymph node enlargement and hemorrhage. While severe neurological symptoms are typically associated with highly virulent PTV 1 strains, other genotypes, including PTV 2, are generally considered asymptomatic. However, an outbreak of PTV 2 in pig farms in Henan Province, China, resulted in severe diarrhea, ataxia, hind limb paralysis, and a mortality rate of up to 38% in piglets. This indicates that certain non-PTV 1 strains may also possess high pathogenicity and lethality. Future research will employ PTV-GXLZ2024, which achieves a viral titer of 10^6.8^ TCID_50_/mL in infected PK-15 cells and consistently induces cytopathic effects, to further investigate PTV pathogenesis and infection mechanisms.

Genetic recombination is integral to the evolutionary dynamics of small RNA viruses, such as PTV. Recombination events, prevalent in the evolutionary history of PTV, facilitate rapid genetic diversification and the emergence of novel genotypes [39]. Frequent recombination events in porcine teschovirus (PTV), particularly inter- and intra-genotypic exchanges, drive viral diversification through preferential clustering in the VP1 capsid gene and non-structural regions [40]. This is exemplified by Hunan surveillance data revealing nine recombination events among 40 strains, collectively accelerating viral evolution and modulating pathogenicity [30]. In this study, we identified a recombination event in the non-structural 2C-3A region of PTV-GXLZ2024 between two local strains. Recombination is an important driver of RNA virus evolution. The genome of PTV-GXLZ2024 underwent exchange in this region. This may enhance viral adaptability and genetic diversity through novel variant emergence [41].

During viral infection, the virus exploits the host cell’s endoplasmic reticulum (ER) for replication, resulting in the accumulation of unfolded and misfolded proteins within the ER lumen. This accumulation disrupts ER homeostasis and induces ERs. Prolonged ERs can activate cellular repair mechanisms or trigger programmed cell death as a countermeasure against viral invasion [23]. The UPR, mediated by the PERK, ATF6, and IRE1-XBP1 pathways, is a vital cellular mechanism for restoring ER homeostasis [42]. In this study, we observed that PTV-GXLZ2024 infection specifically activated the PERK-eIF2α-ATF4 pathway, while the ATF6 and IRE1 pathways remained unaffected. Notably, ATF4 expression gradually decreased during infection, whereas CHOP protein levels remained constant. The observed selective activation pattern is atypical and may indicate a distinct replication strategy employed by PTV. Comparable findings have been documented for the Dengue virus (DENV), which transiently activates the PERK pathway at the onset of infection but subsequently suppresses eIF2α phosphorylation to inhibit apoptosis and facilitate viral replication [43]. CHOP, a critical downstream target of the UPR, is predominantly regulated by the PERK-eIF2α-ATF4 pathway. In contrast to the Japanese encephalitis virus (JEV), which upregulates CHOP via PERK-ATF4 to induce apoptosis [44], the Zika virus (ZIKV) inhibits PERK-mediated UPR to prevent neuronal cell damage and apoptosis [45]. Notably, our findings demonstrate that PTV-GXLZ2024 infection induces eIF2α phosphorylation while concurrently reducing ATF4 expression, suggesting that the virus may inhibit ATF4 transcription and translation to prevent CHOP activation. These might reveal a sophisticated strategy by which PTV hijacks the PERK pathway: Early activation provides translational advantage by suppressing host protein synthesis, while subsequent ATF4 degradation—potentially mediated by viral proteases—abrogates interferon-stimulated gene induction. This mechanism likely extends host cell viability, thereby providing an extended period for viral replication. A parallel phenomenon has been observed in ZIKV-infected A549 cells, where incomplete UPR activation leads to increased CHOP transcription but suppressed CHOP translation, potentially fostering an anti-apoptotic environment conducive to viral replication [46].

The activation of the PERK pathway generally leads to the phosphorylation of eIF2α, resulting in the suppression of global protein translation as a mean to mitigate endoplasmic reticulum (ER) stress. In the context of viral infections, PERK activation can decrease viral protein synthesis, thereby inhibiting viral replication. For example, West Nile virus (WNV) demonstrates enhanced replication and release in PERK-deficient cells compared to wild-type cells [47]. Similarly, our findings indicate that the replication and viral titers of PTV-GXLZ2024 are significantly elevated in PERK-knockdown PK-15 cells relative to wild-type cells, aligning with observations made in WNV.

Due to the unavailability of commercially available antibodies for detecting phosphorylated PERK (p-PERK) in porcine cells, we were unable to ascertain whether PERK phosphorylation initiates the activation of the PERK pathway during PTV-GXLZ2024 infection. We propose that PTV-GXLZ2024 may induce eIF2α phosphorylation either directly or indirectly while concurrently disrupting downstream PERK signaling, thereby preventing the activation of CHOP. This mechanism could potentially extend the survival of infected cells, allowing for an extended period of viral replication. Nonetheless, further investigation is required to elucidate the underlying mechanisms.

## 5. Conclusions

A novel strain of PTV, designated as PTV-GXLZ2024, was isolated from fecal samples collected from fattening pigs at a farm located in Liuzhou, Guangxi. The integrity of the viral particles was verified using transmission electron microscopy, and the biological characteristics, including cellular tropism, were thoroughly characterized. Phylogenetic analysis identified PTV-GXLZ2024 as a local strain belonging to the PTV 2 genotype. Moreover, infection with PTV-GXLZ2024 was observed to activate the UPR via the PERK-eIF2α pathway. Notably, the significant downregulation of ATF4 and the constant expression of CHOP suggested an incomplete UPR. Additionally, si-PERK resulted in enhanced replication of PTV-GXLZ2024, indicating a regulatory role for PERK in the viral replication process. Our findings are expected to establish a foundational understanding of the pathogenic mechanisms underlying PTV and to inform the development of novel antiviral therapeutics.

## Figures and Tables

**Table 1:** Primers sequences used for detection.

Primer.	Sequence of Primer (5′-3′)	Cite by
PDcoV-F	ATCCTCCAAGGAGGCTATGC	[26]
PDcoV-R	GCGAATTCTGGATCGTTGTT	
PEDV-F	TTTTCTAATCATTTGGTCAACG	[27]
PEDV-R	AATACTCATACTAAAGTTGGTGG	
PSV-F	GTGGCGACAGGGTACAGAAGAG	[28]
PSV-R	GTGGCGACAGGGTACAGAAGAG	
PAstV-F	GARTTYGATTGGRCKCGKTAYGA	[29]
PAstV-R	GGYTTKACCCACATNCCRAA	
PTV-F	TGTTGTGTTTAAACACAGAAAT	[30]
PTV-R	TTCAACTGACTATACAAAGTAC	

**Table 2:** Primers sequences used for SYBR real-time RT-PCR primers.

Primer	Sequence of Primer (5′-3′)	Product Size (bp)	Consultation
PTV-VP1-F	TGGCTGACCTGCCTGATAAA	181	This study
PTV-VP1-R	AGGTCAAGTGTCCCATCAGG		
ATF4-F	CCCTTTACGTTCTTGCAAACTC	165	[33]
ATF4-R	GCTTCCTATCTCCTTCCGAGA		
CHOP-F	CTCAGGAGGAAGAGGAGGAAG	133	[33]
CHOP-R	GCTAGCTGTGCCACTTTCCTT		
GAPDH-F	CCTTCCGTGTCCCTACTGCCAAC	103	[33]
GAPDH-R	GACGCCTGCTTCACCACCTTCT		

**Table 3:** Homology analysis of the complete genome andVP1 of PTV-GXLZ2024 compared to other PTV strains.

			Identity%		Structure Protein										Non-Structure Protein					
Reference Strain	Country	GeneBank Accession No.	Complete Genome		P1		Vp1		Vp2		Vp3		Vp4		L		P2		P3	
			nt	aa	nt	aa	nt	aa	nt	aa	nt	aa	nt	aa	nt	aa	nt	aa	nt	aa
PTV A1/CHN-NP1-2016	China	MF967586	83.4	76.1	71.3	76.1	69.1	70.2	80.2	82.8	70.0	75.2	83.8	93.2	89.1	95.3	87.3	96.3	91.3	98.8
PTVA2/GX2020	China	OM281048	90.2	93.6	90.6	97.1	89.6	96.6	84.3	85.0	91.9	96.7	89.1	100	90.3	93	86.8	97.1	91.3	98.8
PTV A3/HuN4	China	MF170908	83.0	79.2	71.3	76.8	66.7	71.0	81.1	83.9	71.6	76.9	88.3	98.6	88.4	97.7	87.7	97.3	90.5	98.2
PTV A4/HuN23	China	MF170927	86.8	85.5	79.8	85.4	76.9	79.1	82.5	86.1	80.2	88.0	85.6	100	84.9	88.4	88.9	98.2	92.3	99.4
PTV A5/HuN33	China	MF170937	83.5	80.2	73.9	78.3	68.3	69.5	82.4	86.1	75.0	78.1	85.6	97.3	80.2	88.4	87	98.2	91.3	98.4
PTV A6/HuN25	China	MF170929	86.8	85.7	80	86.9	78.5	82.6	83.9	85.7	80.0	89.7	87.4	100	88.4	93	88	97.5	92.1	99.4
PTV A7/F_43	USA	AF296092	81.8	76.3	78.8	77.3	73.0	75.6	77.3	82.9	74.7	76.9	83.3	97.3	87.2	95.3	84.6	95.9	86.8	97.4
PTV A8/Fuyu2009-2	China	KC757344	85.0	82.5	73.6	86.4	75.6	80.3	82.6	86.1	80.4	90.9	87.4	100	85.3	89.5	83.9	95.3	90.6	98.8
PTV A9/HuN1	China	MF170905	83.5	77.6	72.7	76.8	70.9	73.6	80.3	81.3	71.9	78.9	83.3	94.6	90.3	94.2	85.7	96.7	91.7	99
PTV A10/Vir_46188	Germany	AF296119	81.4	79.1	70.1	74.8	64.5	67.6	80.5	82.1	72.3	76.4	82.9	95.9	87.6	93	84.4	97.3	89.2	98.7
PTV A11/HuN19	China	MF170923	83.7	79.3	72.1	76.9	67.3	71.8	80.1	83.9	72.7	77.3	87.4	97.3	91.5	97.7	88	97.1	91.8	98.8
PTV B1/HuN41	China	MG875515	71.6	51.3	62.8	64.8	54.8	52.9	69.7	66.7	63.6	66.9	82.0	97.3	73.3	73.3	79.2	90.2	74	83.4
PTV B3/HuN42	China	MG875516	72.1	54.7	64.1	65.2	57.2	51.7	70.4	67.9	65.4	70.7	82.4	94.6	71.3	72.1	78.8	89.9	73.9	83.6
PTV B22/JiangX1	China	MN094632	73.2	55.9	67.3	71.9	64.4	65.2	74.3	76.1	67.6	74.8	76.6	90.5	69.4	73.3	79	89.5	74.2	84

## Data Availability

The complete genome sequences of PTV-GXLZ2024 were deposited in NCBI’s Genbank and are available via accession number PQ358532.1. The data sets reported in this study will be accessible from the corresponding author upon reasonable request.
